# MTDH associates with m6A RNA methylation and predicts cancer response for immune checkpoint treatment

**DOI:** 10.1016/j.isci.2021.103102

**Published:** 2021-09-09

**Authors:** Fen Zhang, Huimei Huang, Yuexiang Qin, Changhan Chen, Li She, Juncheng Wang, Donghai Huang, Qinglai Tang, Yong Liu, Gangcai Zhu, Xin Zhang

**Affiliations:** 1 Department of Emergency Medicine, Changsha Central Hospital, University of South China, Changsha 410001, China; 2 Department of Otolaryngology-Head and Neck Surgery, Second Xiangya Hospital, Central South University, Changsha 410010, China; 3 Health Management Center, Third Xiangya Hospital, Central South University, Changsha 410011, China; 4 Department of Otolaryngology-Head and Neck Surgery, Xiangya Hospital, Central South University, Changsha 410008, China

**Keywords:** Biological sciences, Molecular biology, Expression study

## Abstract

Immune checkpoint blockade (ICB) persistently provides a prognosis improvement but only in a small fraction of patients with cancer due to immunotherapy resistance induced by the consecutive activated oncogenic pathways, including MAPK, Akt, and WNT pathway partially driven by Metadherin (MTDH). However, there is no study to investigate the potential role and mechanisms of MTDH in ICB-treated cancers. Here, we systematically explored the cohorts from The Cancer Genome Atlas (TCGA) and independent cancer cohorts. Elevated MTDH expression was founded to associate with a worse overall survival and poorer immune response in patients with cancer. Dysregulated tumor-infiltrating immune cells and inhibitory immune checkpoint expression were correlated with MTDH expression. Furthermore, the mutual interactions between epithelial-to-mesenchymal-transition, m6A-RNA-methylation, and MTDH may illustrate the potential mechanisms of MTDH resistant to ICB treatment. Although more designed experiments and trials are needed in the future, targeting MTDH may help to overcome immunotherapy resistance in a wide range of cancers.

## Highlights

• MTDH associates with prognosis and immunotherapy response for patients with cancer
• MTDH associates with dysregulated tumor immune environment and checkpoint expression
• The MTDH/m6A RNA methylation/EMT pathway may contribute to immunotherapy resistance

## Graphical abstract

**Figure:**
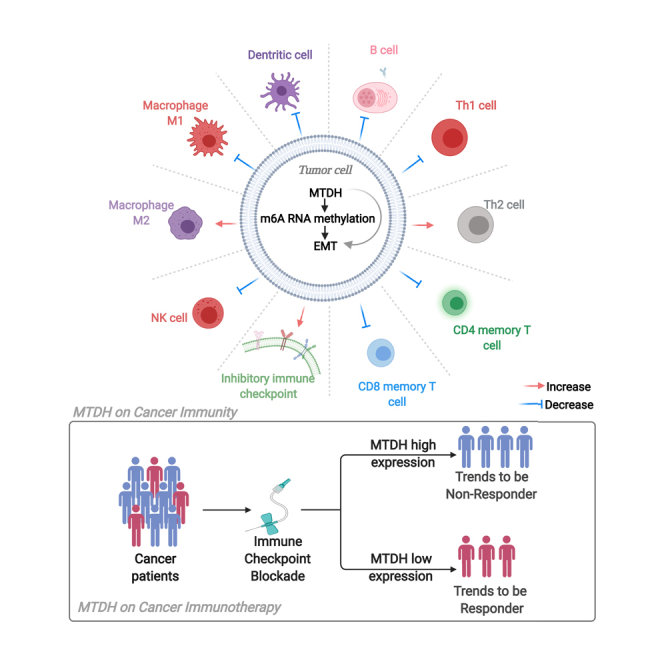

## Introduction

Cancer ranks as a leading cause of death and a massive barrier to increasing life expectancy globally in decades (Sung et al., 2021). According to World Health Organization statistics, cancer has accounted for nearly 10 million deaths in 2020, which results in countless economic costs and social burdens. Surgery and chemoradiotherapy have been considerably prolonged life of patients with cancer for many decades. However, the improvement of overall outcome for patients with cancer hit a plateau until immune checkpoint blockade (ICB) developed in recent years. ICB, represented by anti-PD1/PD-L1 and anti-CTLA4, has been undoubtedly revolutionized the remedy for various types of cancer by the unprecedented extent of clinical responses. Reports of patients with cancer achieving complete remissions are accumulating, be that as it may, the proportion of non-responders to ICB treatment remains in major. Efforts on the mechanism of immunotherapy resistance are increasing to assist physicians with the responder-candidate selection before treatment. Except for immunosuppressive microenvironment (such as Tregs and MDSCs) and insufficient tumor immunogenicity (such as impaired dendritic cell and MHC dysfunction), activations of oncogenic pathways, including MAPK, PI3K, and WNT/β-catenin pathway, are reported to drive carcinogenesis and ICB resistance (Lei et al., 2020). Therefore, tumor-intrinsic oncogenic pathways for PD1/PD-L1 blockade resistance should not be undermined (Lei et al., 2020).

Metadherin (MTDH), first identified in primary human fetal astrocytes by rapid subtraction hybridization and named AEG-1, is a well-known oncogene in various cancers, including head and neck cancer, lung cancer, liver cancer, breast cancer, and melanoma (Emdad et al., 2016; Robertson et al., 2018; Shen et al., 2021; Su et al., 2002; Yu et al., 2014; Zhu et al., 2017). MTDH promotes multiple hall markers of aggressive cancer behaviors, including tumor proliferating, metastasis, angiogenesis, and chemotherapy resistance, by activating MAPK, PI3K/AKT, and WNT/β-catenin pathways in diverse cancers (Manna and Sarkar, 2021; Yoo et al., 2011). Theoretically speaking, the expression of MTDH in cancer would be dysregulated and might impact ICB immunotherapy response for patients with cancer. However, there is no study to investigate the role of MTDH in cancer with ICB treatment. Here, we systematically investigated the expression of MTDH, deconvoluted tumor immune environments, and estimated gene signatures in the The Cancer Genome Atlas (TCGA) pan-cancer, independent cancer series, and multiple cancer immunotherapy cohorts. High expression of MTDH was discovered and validated to predict a worse overall survival for immunotherapy-naïve and immunotherapy-treated patients with cancer. Furthermore, the connections of epithelial-to-mesenchymal transition (EMT) and m6A RNA methylation with MTDH may illustrate the potential immune-related mechanisms underlying MTDH resistance to ICB treatment and unfavorable prognosis.

## Results

### Elevated expression of MTDH in diverse types of cancer

**Figure 1:**
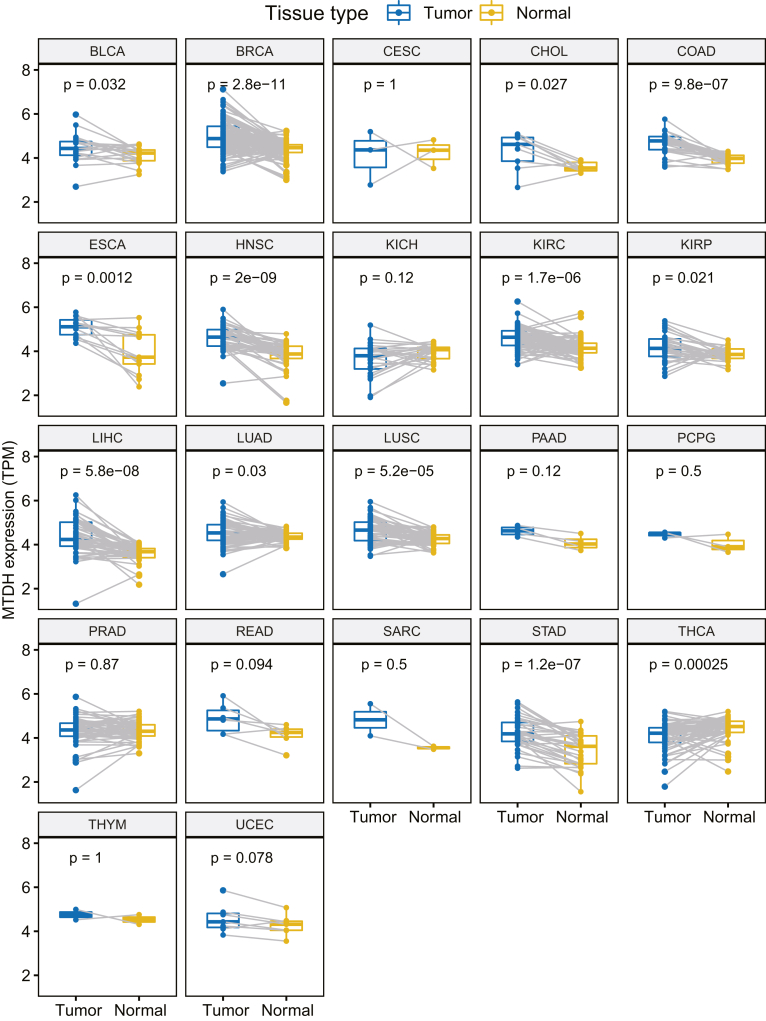
The pairwise comparisons of MTDH mRNA expression in tumor and paired normal tissues across 22 types of cancer The boxplots show that MTDH mRNA is significantly overexpressed in tumor over normal tissues within 12 cancer types including BLCA, BRCA, CHOL, COAD, ESCA, HNSC, KIRC, KIRP, LIHC, LUAD, LUSC, and STAD (all p < 0.05). Wilcox test was used in the comparison. BLCA: bladder urothelial carcinoma, BRCA: breast invasive carcinoma, CESC: cervical squamous cell carcinoma and endocervical adenocarcinoma, CHOL: cholangiocarcinoma, COAD: colon adenocarcinoma, ESCA: esophageal carcinoma, HNSC: head and neck squamous cell carcinoma, KIRC: kidney renal clear cell carcinoma, KIRP: kidney renal papillary cell carcinoma, LIHC: liver hepatocellular carcinoma, LUAD: lung adenocarcinoma, LUSC: lung squamous cell carcinoma, STAD: stomach adenocarcinoma, THCA: thyroid carcinoma, PAAD: pancreatic adenocarcinoma, PCPG: pheochromocytoma and paraganglioma, PRAD: prostate adenocarcinoma, READ: rectum adenocarcinoma, SARC: sarcoma, THYM: thymoma, UCEC: uterine corpus endometrial carcinoma. See also Figure S1.

To systematically investigate the mRNA expression of MTDH in pan-cancer levels, we performed the pairwise comparisons of MTDH mRNA in tumor samples and paired-adjacent normal tissues in the available 22 kinds of cancers from the TCGA dataset. As shown in Figure 1, MTDH expression was significantly decreased in THCA (thyroid carcinoma) but elevated in 12 kinds of tumors as compared to their adjacent normal tissues from bladder urothelial carcinoma (BLCA), breast invasive carcinoma (BRCA), cholangiocarcinoma (CHOL), colon adenocarcinoma (COAD), esophageal carcinoma (ESCA), head and neck cancer (HNSC), liver hepatocellular carcinoma (LIHC), kidney renal papillary cell carcinoma (KIRC), kidney renal clear cell carcinoma (KIRP), lung adenocarcinoma (LUAD), lung squamous cell carcinoma (LUSC), and stomach adenocarcinoma (STAD) (all p < 0.05). Furthermore, the upregulated MTDH protein expressions were confirmed in multiple independent proteomics datasets (all p < 0.05, Figure S1).

### MTDH expression predicts an unfavorable overall survival in cancer

**Figure 2:**
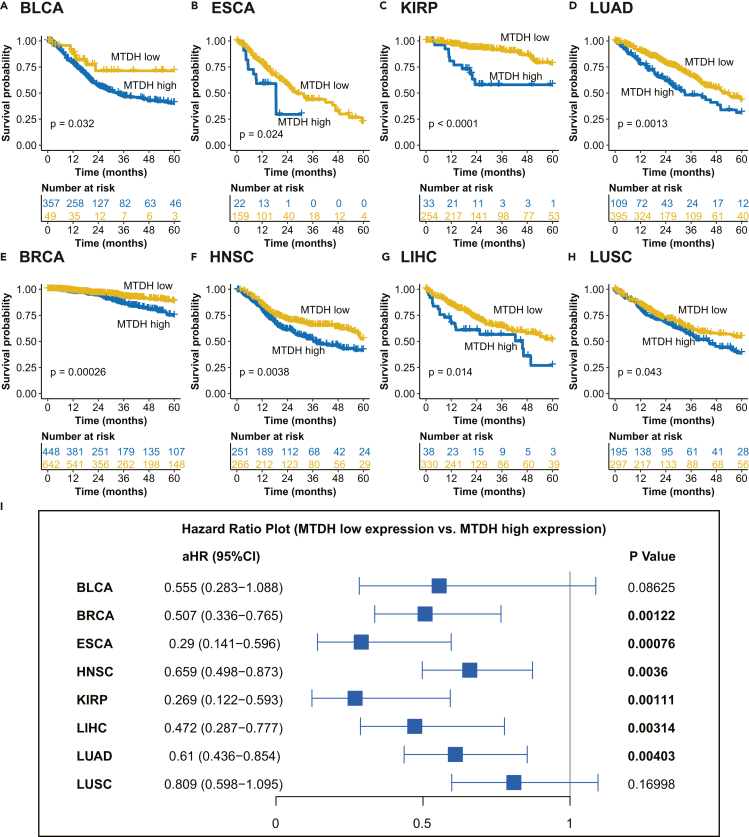
High expression of MTDH associates with a poor overall survival in various of cancers (A–H) Kaplan-Meier survival analysis displaying in overall survival between patients with cancer with high and low MTDH expression; log rank was used for p value calculation. BLCA: bladder urothelial carcinoma, ESCA: esophageal carcinoma, KIRP: kidney renal papillary cell carcinoma, LUAD: lung adenocarcinoma, BRCA: breast invasive carcinoma, HNSC: head and neck squamous cell carcinoma, LIHC: liver hepatocellular carcinoma, LUSC: lung squamous cell carcinoma. (I) The hazard ratio of dead in MTDH low expression patients vs. MTDH high expression patients across various types of cancer. aHR: hazard ratio with 95% confidence interval from Cox proportion hazard models adjusted for TNM stage. See also Figure S2.

Since higher expression of MTDH was found in cancer, we explored the prognostic value of MTDH expression in the above 12 kinds of cancer. As shown in Figures 2A–2H, high expression of MTDH indicated a significantly poorer overall survival in BLCA, ESCA, KIRP, LUAD, BRCA, HNSC, LIHC, and LUSC. After adjusted for TNM stages, multivariate Cox proportional hazards analysis showed that lower expression of MTDH had decreased hazard ratios of death in BRCA, ESCA, HNSC, KIRP, LIHC, and LUAD (Figure 2I).

To validate the above findings, independent GEO cohorts were applied to analyze the associations of MTDH expression with overall survival in cancer. High expression of MTDH remained an unfavorable overall survival indicator in these independent cohorts (p < 0.01, Figure S2).

### MTDH expression negatively correlates with immunotherapy response in cancer

Previous studies have shown that tumor mutation burden (TMB), fPD1, and tumor immunogenicity score can correlate with the objective response for ICB treatment in TCGA pan-cancer (Lee and Ruppin, 2019; Wang et al., 2019; Yarchoan et al., 2017). Here, we used the same strategies to evaluate the correlation of objective response to anti-PD1/PD-L1 with MTDH expression in the cancers whose overall survival correlated with MTDH expression in the above result.

**Figure 3:**
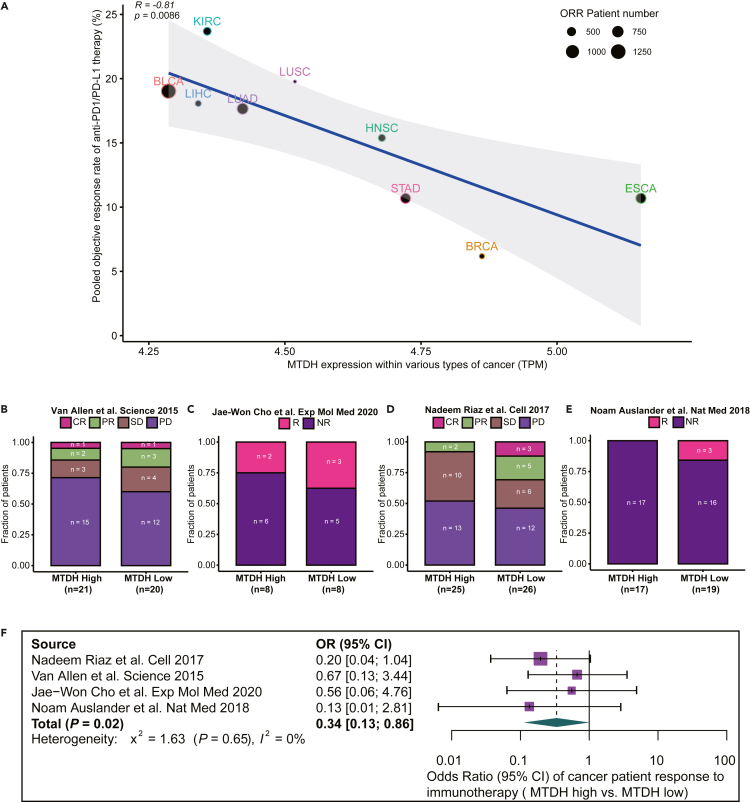
High expression of MTDH in cancer may associate with immunotherapy resistance (A) The Pearson correlation of MTDH expression with objective response rate (ORR) to anti-PD1/PD-L1 therapy across cancer types. The MTDH expression was represented by its median value within the cancer type. ORR was pooled by the current trial data. (B–E) Four independent datasets show the proportion of immunotherapy responders between the MTDH high group and MTDH low group. (F) Meta-analysis on the above four independent studies present that high expression of MTDH in patients with cancer have significantly lower possibilities to response to immunotherapy (p = 0.02). CR: complete response, PR: partial response, SD: stable disease, PD: progressive disease, R: responder, NR: non-responder, detailed definitions were mentioned in STAR methods section, OR: odds ratio. BLCA: bladder urothelial carcinoma, ESCA: esophageal carcinoma, KIRC: kidney renal clear cell carcinoma, LUAD: lung adenocarcinoma, BRCA: breast invasive carcinoma, HNSC: head and neck squamous cell carcinoma, LIHC: liver hepatocellular carcinoma, LUSC: lung squamous cell carcinoma, STAD: stomach adenocarcinoma.

As shown in Figure 3A, the pooled objective response rate for anti-PD-1 or anti-PD-L1 therapy against the corresponding median MTDH expression across multiple cancer types was plotted. Moreover, there was a significant negative correlation between the MTDH expression and the objective response rate (Pearson coefficient: R = −0.81; p < 0.001; Figure 3A), which indicated that high expression of MTDH might associate with poor immunotherapy response in cancer.

We further analyzed four independent cancer cohorts to confirm the negative correlation of MTDH expression with immunotherapy response. As shown in Figures 3B–3E, patients with cancer and MTDH-high expression trended to be non-responder or progressive disease (PD). Low expression of MTDH was more likely to be a complete response or partial response. Meta-analysis for above four cohorts showed that the immunotherapy response rate in the MTDH-high expression group was significantly lower than that in the MTDH-Low expression group (the pooled odds ratio = 0.34, 95%CI = 0.13–0.86, p = 0.02, Figure 3F).

### MTDH associates with tumor-infiltrating immune cells and immune checkpoint expression

**Figure 4:**
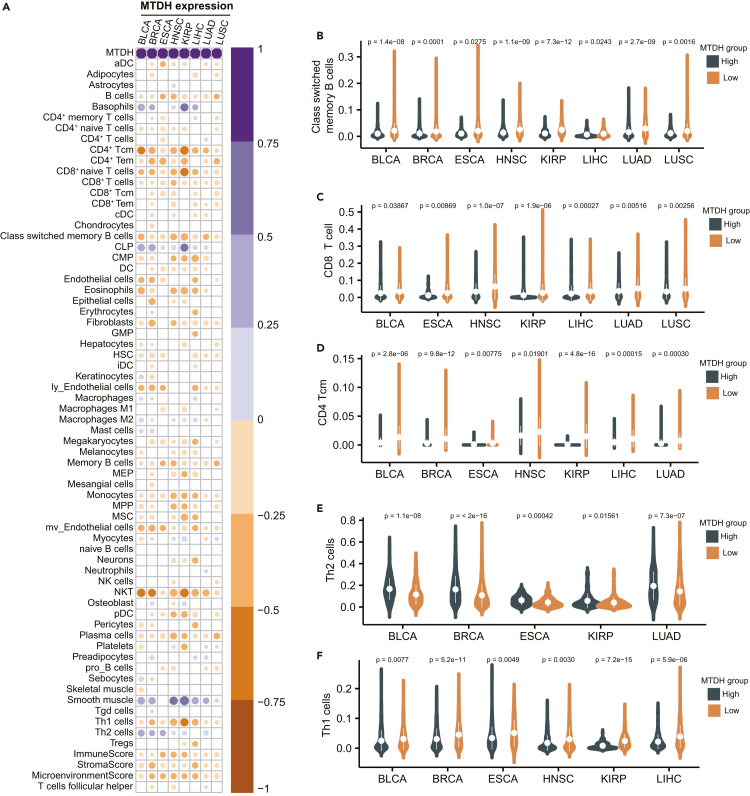
The correlation of MTDH expression and immune cells estimation in cancer (A) The colored cycles shows the Pearson correlations of MTDH expression with immune cell and component estimation deconvoluted by xCell or CIBERSORT in BLCA, BRCA, ESCA, HNSC, KIRP, LIHC, LUAD, and LUSC; the cycled block presents into white if p value less than 0.05. The depth of color represents the extent of Pearson correlation. (B**–**F) High expression of MTDH in tumors indicates lower class switched memory B cells, Th1 cells, CD4+ central memory T cells (CD4 Tcm cells), CD8+ T cells (CD8 T cells), and higher Th2 cells as compared to the tumors with MTDH low expression. BLCA: bladder urothelial carcinoma, ESCA: esophageal carcinoma, KIRP: kidney renal papillary cell carcinoma, LUAD: lung adenocarcinoma, BRCA: breast invasive carcinoma, HNSC: head and neck squamous cell carcinoma, LIHC: liver hepatocellular carcinoma, LUSC: lung squamous cell carcinoma. See also Figure S3.

Immunotherapy response will be determined by tumor-infiltrating immune cells. To discover the potential mechanism of MTDH’s role in immunotherapy, we deconvoluted the tumor immune microenvironment across the above cancers. The significant correlations of MTDH expression with estimated scores of tumor-infiltrating immune cells were visualized in a heatmap (Figure 4A). The class-switched memory B cells were significantly lower in the MTDH-high group comparing to the MTDH-low group (p < 0.05, Figure 4B). The estimated score of CD8^+^ T cells, CD4^+^ central memory T cells (CD4^+^ Tcm), and Th1 cells were increased significantly in MTDH-low expression patients across diverse cancer types (Figures 4C–4E). Conversely, the suppressive immune cell, like the proportion of Th2 cells, was higher in MTDH-high patients than in MTDH-low patients (Figure 4F).

Except for tumor-infiltrated immune cells, the expressions of co-inhibitory immune checkpoints were explored as well. MTDH expression positively correlated with almost all the current known co-inhibitory immune checkpoints, including PD-L1/L2, CTLA4, TIM-3, and TIGIT, across BLCA, BRCA, ESCA, HNSC, KIRP, LIHC, LUAD, and LUSC (Figure S3A). Besides, there was no significant difference in neoantigen number or TMB between MTDH-high and MTDH-low expressed cancers in LUAD, BLCA, KIRP, LIHC, and HNSC (p > 0.05, Figures S3B and S3C).

### Epithelial-to-mesenchymal transition may involve molecular functions of MTDH

**Figure 5:**
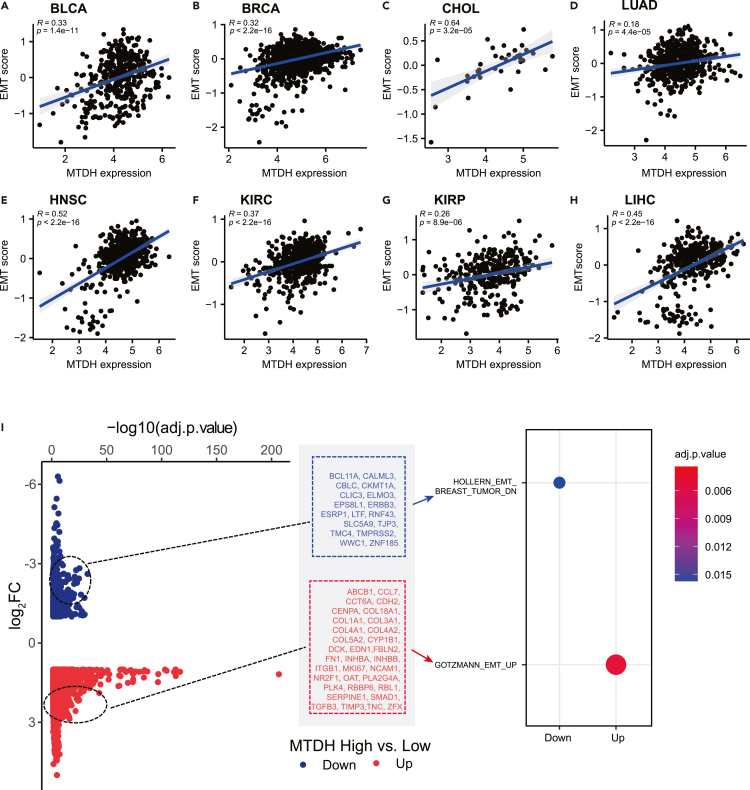
MTDH expression associates with epithelial-mesenchymal transition (EMT) in cancer (A–H) The expression of MTDH mRNA significantly correlates with estimated score of EMT within tumor samples from BLCA, BRCA, CHOL, LUAD, HNSC, KIRC, KIRP, and LIHC (all p < 0.05). (I) The significantly differential gene expressions in the group of MTDH-high samples compared to the samples with MTDH-low expression, which enriched into EMT pathway by MSigDb enrichment analysis. BLCA: bladder urothelial carcinoma, BRCA: breast invasive carcinoma, CHOL: cholangiocarcinoma, HNSC: head and neck squamous cell carcinoma, KIRC: kidney renal clear cell carcinoma, KIRP: kidney renal papillary cell carcinoma, LIHC: liver hepatocellular carcinoma, LUAD: lung adenocarcinoma, Up: upregulated genes in MTDH-high vs. MTDH-low, Down: downregulated genes in MTDH-high vs. MTDH-low, adj. p.value: adjusted p value by Benjamini-Hochberg (BH) method. See also Figure S4.

Numerous studies and our previous work showed that high expression of MTDH promoted EMT in head and neck cancers (Emdad et al., 2016; Yu et al., 2014). Therefore, the associations of EMT and MTDH were investigated. As shown in Figures 5A–5H, there are significant positive correlations between MTDH expression and the estimated scores of EMT across BLCA, BRCA, CHOL, LUAD, HNSC, KIRC, KIRP, and LIHC.

More importantly, the differentially expressed genes comparing MTDH-high to MTDH-low tumors were discovered based on RNA-seq expression profiles (Figure 5I). These differential genes included EMT-related genes such as SMAD1, TGFB3, NCAM1, etc., which could be significantly enriched in EMT-up and EMT-down pathways by MSigDb enrichment analysis (Figure 5I). Taken together, the downstream genes of MTDH in cancer may associate with EMT. What more, the KEGG and GO enrichment assay indicated that MTDH downstream genes also enriched in integrin and collagen binding and PI3K-Akt pathway (Figure S4), which illustrates the potential mechanism of MTDH regulating EMT in a cancer cell.

### m6A RNA methylation correlates with MTDH expression in cancer

**Figure 6:**
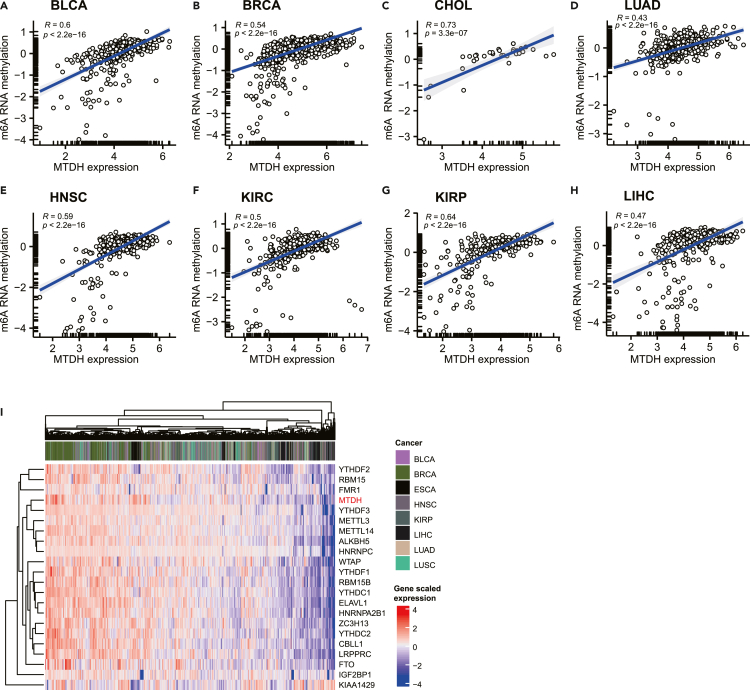
MTDH expression associates with the estimation of m6A RNA methylation in cancer (A–H). The expression of MTDH mRNA significantly correlates with estimated score of m6A RNA methylation within tumor samples from BLCA, BRCA, CHOL, LUAD, HNSC, KIRC, KIRP, and LIHC (all p < 0.05). (I) The heatmap shows the gene expression pattern between MTDH and m6A RNA methylation-associated genes; BLCA: bladder urothelial carcinoma, BRCA: breast invasive carcinoma, CHOL: cholangiocarcinoma, HNSC: head and neck squamous cell carcinoma, KIRC: kidney renal clear cell carcinoma, KIRP: kidney renal papillary cell carcinoma, LIHC: liver hepatocellular carcinoma, LUAD: lung adenocarcinoma.

To further investigate the possible mechanism of MTDH regulating EMT, given that m6A RNA methylation is crucial for promoting EMT in cancer (Chen et al., 2020; Jin et al., 2020; Lin et al., 2019; Ma et al., 2021; Xu et al., 2021), the correlation between m6A RNA methylation and MTDH was performed. As shown in Figures 6A–6H, the estimated score of m6A RNA methylation was significantly correlated with MTDH expression in BLCA (R = 0.6, p < 0.01), BRCA (R = 0.54, p < 0.01), CHOL (R = 0.73, p < 0.01), LUAD (R = 0.43, p < 0.01), HNSC (R = 0.53, p < 0.01), KIRC (R = 0.5, p < 0.01), KIRP (R = 0.64, p < 0.01), and LIHC (R = 0.47, p < 0.01).

Furthermore, the expression pattern of MTDH in the above cancers showed similar trends with individual m6A RNA methylation genes such as METTL3 and METTL14 (Figure 6I). Therefore, the role of MTDH in cancer may connect with the function of m6A RNA methylation.

### The mutual interactions between EMT, m6A RNA methylation, and MTDH in cancer

**Figure 7:**
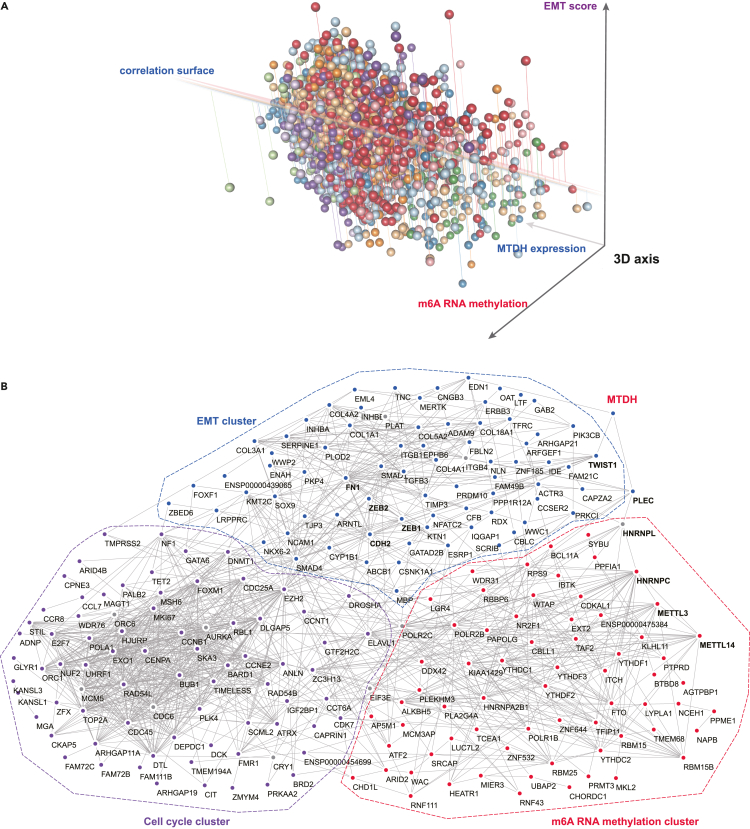
The correlation and connections between MTDH, EMT, and m6A RNA methylation in cancer (A) 3D axis shows the mutual correlations between MTDH expression, EMT score, and m6A RNA methylation signature score in cancer (all p < 0.05), each sphere means a tumor sample, different color represent cancer source which includes BLCA, BRCA, CHOL, LUAD, HNSC, KIRC, KIRP, and LIHC. (B) The comprehensive protein-protein interaction networks between EMT signature, m6A RNA methylation, and the most differently expressed genes in MTDH-high tumors comparing to MTDH-low tumors were constructed in String database and re-visualized by Cytoscape.

Both EMT and m6A RNA methylation correlated with MTDH expression in cancers; therefore, we hypothesized a mutual correlation between MTDH, EMT, and m6A RNA methylation. As shown in Figure 7A, the 3D plot showed the mutual correlations between EMT, m6A RNA methylation, and MTDH expression in cancer. Moreover, the protein-protein interaction network including m6A RNA methylation-associated genes, EMT-associated genes, and the most MTDH-associated differently expressed genes showed a comprehensive connection between MTDH, EMT, and m6A RNA methylation in multiple molecular levels (Figure 7B), which indicated that a molecular pathway from MTDH to m6A RNA methylation and EMT might exist in cancers.

### The estimated score of MTDH/m6A RNA methylation/EMT pathway in cancer immunotherapy

In order to discover the significance of the comprehensive pathway connecting with MTDH, EMT, and m6A RNA methylation, we estimated the score of the pathway by z-scored their associated genes in TCGA tumors and an independent cancer immunotherapy cohort.

**Figure 8:**
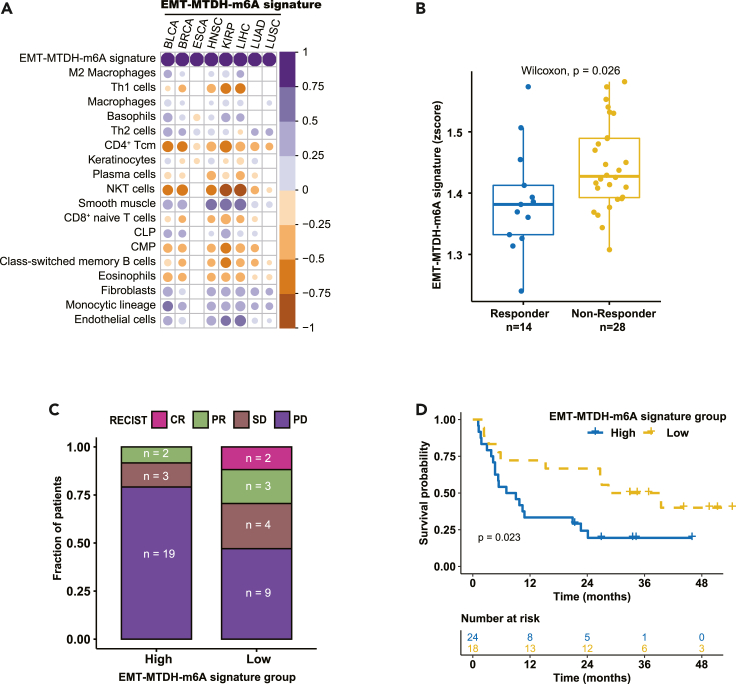
High estimated EMT-MTDH-m6A signature correlates with immune cells and resistance to immunotherapy (A) Pearson correlations of EMT-MTDH-m6A signature with immune cells in BLCA, BRCA, ESCA, HNSC, KIRP, LIHC, LUAD, and LUSC; the cycled block presents into white if p value less than 0.05. The depth of color represents the extent of Pearson correlation. (B) The estimated EMT-MTDH-m6A signature was significantly higher in immunotherapy non-responders as compared to immunotherapy responders. (C) Different immunotherapy responses between patients with high EMT-MTDH-m6A signature and patients with high EMT-MTDH-m6A signature. (D) Kaplan-Meier curve indicates EMT-MTDH-m6A signature is an unfavorable overall survival factor (p = 0.023).

Immunosuppressive cells (such as M2 macrophages, Th2 cells, fibroblasts) positively correlated with the MTDH/m6A/EMT pathway score across diverse cancers. In contrast, CD4^+^ Tcm, NKT cells, Th1 cells, and CD8^+^ naive T cells negatively correlated with the MTDH/m6A/EMT pathway (all p < 0.05, Figure 8A). Compared to immunotherapy responders, there was a significantly higher MTDH/m6A/EMT pathway score in non-responders (p = 0.026, Figure 8B). All the complete response patients (n = 2) were in MTDH/m6A/EMT pathway score low group (Figure 8C). More patients with progressive disease after immunotherapy were founded in the high-MTDH/m6A/EMT pathway score group than their low counterparts (n = 19 vs. n = 9, Figure 8C). Furthermore, the Kaplan-Meier curve indicates that high-MTDH/m6A/EMT pathway score was an unfavorable overall survival factor in the immunotherapy-treated patients with cancer (p = 0.023, Figure 8D).

## Discussion

Our study is the first investigation focusing on the associations between MTDH expression and immunotherapy response in multiple cancer cohorts, which consistently indicated that high expression of MTDH might resist cancer immunotherapy. The immune response is determined by the interactions between tumor cells and the immune environment. The mechanism of resistance to immunotherapy is obscure, but both tumor-cell-intrinsic and tumor-cell-extrinsic factors may be involved (Sharma et al., 2017).

Multiple tumor-intrinsic mechanisms, including MAPK, PTEN/PI3K, WNT/beta-catenin, and IFN signaling pathways, have recently been identified to be related to immunotherapy resistance (Sharma et al., 2017). Concretely, activation of the MAPK pathway could result in secreted VEGF and IL-8, which would inhibit T cell recruitment and function (Liu et al., 2013). Similarly, PTEN loss in cancer, which enhances PI3K signaling, was associated with resistance to immune checkpoint therapy (Peng et al., 2016). Constitutive WNT/beta-catenin pathway may associate with decreased expression of CCL4, a chemokine that attracts CD103^+^ DCs, and ineffective response to immune checkpoint therapy (Spranger et al., 2015).

Our previous work and other colleagues’ studies showed that MTDH is an oncogene and a trigger that activates PI3K, MAPK, and WNT pathway in head and neck cancer and other types of cancer (Emdad et al., 2016; Robertson et al., 2018; Yoo et al., 2011; Yu et al., 2014), which indicates MTDH would serve as an unfavorable prognosis indicator for patients with cancer. This study confirmed that MTDH expression is significantly increased in various tumors than paired adjuvant normal tissues except for thyroid cancer. The downregulated expression of MTDH in thyroid cancer was in contrast to the previous thyroid cancer publication (Moore et al., 2016). We excluded thyroid cancer from our downstream analysis for conscientiousness. After adjusting for TNM stages, MTDH expression was associated with overall survival time for patients with BRCA, ESCA, HNSC, KIRP, LIHC, or LUAD. More importantly, based on the above tumor-cell-intrinsic mechanism of immunotherapy resistance, high expression of MTDH may theoretically associate with poor immunotherapy response due to its regulations on PI3K, MAPK, and WNT pathways. The negative correlation of MTDH expression and pooled response rate of immune checkpoint blockade therapy is significant among various cancer types in our preliminary TCGA analysis. More importantly, we confirmed the unfavorable role of MTDH expression on immunotherapy response in four independent clinical trials (Auslander et al., 2018; Cho et al., 2020; Riaz et al., 2017; Van Allen et al., 2015). Beyond that, we further discovered the additional underlying mechanisms, including tumor-cell-extrinsic factors.

Tumor-cell-extrinsic mechanisms of immunotherapy resistance involve components other than tumor cells within the tumor microenvironment, including increased distribution of Th2 cells, M2 macrophages, and decreased tumor-infiltrating CD8^+^ T cells, DCs, Th1 cells, CD4^+^ T cells, and M1 macrophages (Aldea et al., 2021). This study found that MTDH expression positively correlated with Th2 cells and M2 macrophages, which indicates that patients with high expression of MTDH would recruit more Th2 cells and M2 macrophages. Th2 cells secrete cytokines such as IL-10, IL-4, and IL-5, which inhibit cytotoxic CD8^+^ T cell proliferation and promote macrophage polarization to M2 type (Liu et al., 2009). M2 macrophages express higher levels of anti-inflammatory cytokines that skewing tumor microenvironment into immunosuppressive (Yang and Zhang, 2017). Our study consistently showed that high expression of MTDH in diverse cancers is likely to be a lower enrichment for Th1 cells, B cells, and CD4/CD8 memory cells (including central memory and effector memory T cell), involved in the immune response ejection and maintenance to viral or tumor antigens (Bagarazzi et al., 2012; Egelston et al., 2021; Helmink et al., 2020; Sharma et al., 2017). These correlations of MTDH expression and immune cell infiltration may explain the association between high expression of MTDH and inadequate immunotherapy response.

Interestingly, we found that MTDH expression positively correlated with many inhibitory immune checkpoints, including PD-1/PD-L1, PD-L2, Tim-3, TIGIT, and LAG3, which demonstrated that a single or few immune checkpoint blockades might not be sufficient to recharge cytotoxic T cell killings due to the high expression of other inhibitory checkpoints (He and Xu, 2020). Several emerging trials are undergoing to test the efficiency of immune checkpoint blockade combination in patients with cancer. Here, our study indicates that combining with MTDH inhibition rather than the combination of alternative immune checkpoint blockades should be suggested to overcome immunotherapy resistance for patients with elevated MTDH expression. TMB was approved as an indicator for immunotherapy by U.S. Food and Drug Administration (FDA). Neoantigen number is crucial for the recognition and killing capabilities of CD8^+^ T cells. Therefore, the relationship between MTDH expression and TMB or neoantigen was investigated in this study. There is no significantly different TMB or neoantigen between low and high expressed MTDH patients, indicating that the mechanism of MTDH-associated immunotherapy resistance might be independent of tumor mutation burden and neoantigen numbers.

The EMT in cancer is a process of dedifferentiation due to external stimulation like TGF-β or IL-8, activation of transcription factors including Snail, Slug Twist, Zeb1, and Zeb2 (Horn et al., 2020), or oncogenic signals like WNT, Notch, and MAPK pathways (Stemmler et al., 2019). EMT confers cancer cells with increased tumor metastatic potential and more resistance to immunotherapy (Dongre and Weinberg, 2019; Horn et al., 2020). This study found that the positive correlation of MTDH expression and estimated EMT score is significant in various cancers. What more, the differentially expressed genes between MTDH-high and MTDH-low tumor samples include EMT-associated genes, such as fibronectin (FN1), N-cadherin (CDH2), and SMAD. These findings imply the possible role of EMT in MTDH-associated immunotherapy resistance.

Current preclinical and clinical research demonstrates that EMT correlates with increased resistance to immunotherapy (Horn et al., 2020). Hugo et al. reported a high transcriptomic signature of EMT-related genes associated with primary resistance to anti-PD-1 therapy in patients with metastatic melanoma (Hugo et al., 2017). EMT-associated transcription factors and downstream pathways that drive tumor cell metastasis also help confer resistance against antitumor immunity and therapy. For example, the overexpression of Snail was shown to induce Tregs and to inhibit dendritic cell function through the secretion of TGF-β and TSP1 in melanoma (Kudo-Saito et al., 2009). Furthermore, Snail was reported to shield tumor cells from T cell-mediated lysis via autophagy (Akalay et al., 2013). A recent study showed that hypoxic conditions in the lung cancer environment could upregulate numerous EMT-associated transcription factors, including Snail and Twist, resulting in highly mesenchymal cancer cells resisting T cell and NK cell-mediated killing (Terry et al., 2017). The expressions of inhibitory immune checkpoints, like PD-L1, were elevated on the cancer cell or immune cells as epithelial cancer cells transiting to mesenchymal cancer cells (Horn et al., 2020; Qin et al., 2020). Therefore, EMT is an intermediary phenomenon connecting with tumor-cell-intrinsic and tumor-cell-extrinsic mechanisms of immunotherapy resistance. Taken together, MTDH may promote immunotherapy resistance by regulating the EMT of the cancer cell. The mechanism of MTDH regulating EMT remains unknown; our previous data showed PI3K/Akt might involve in the MTDH-induced EMT process (Yu et al., 2014).

m6A RNA methylation is crucial for the regulation of EMT through WNT/beta-catenin, PI3K/Akt, LAST2/YAP pathway, or Sox4 mRNA stability (Chen et al., 2020; Jin et al., 2020; Lin et al., 2019; Ma et al., 2021; Xu et al., 2021). Our study showed that the most differential genes in the high expression of MTDH tumor might be enriched in the activation of the PI3K/Akt pathway, indicating MTDH may regulate PI3K/Akt-induced EMT by m6A RNA methylation. Analogous to histones and DNA, abundant eukaryotic mRNAs can also be reversibly modified by methylation on m6A sites (Roundtree et al., 2017). m6A modification is mainly mediated by a methyltransferase (named as writers including METTL3, METTL14, KIAA1429, and WTAP), m6A demethylases (erasers) consists of ALKBH5 and FTO, and m6A modification reader proteins mainly include YTHDF1/2/3, IGF2BP1/2/3, HNRNPA2B1, and HNRNPC (Roundtree et al., 2017). Interestingly, m6A RNA methylation was reported to regulate T cell-mediated pathogenesis (Li et al., 2017a) and immunotherapy sensitivity in colorectal cancer (Wang et al., 2020) and melanoma (Li et al., 2020; Yang et al., 2019) and implied that the positive correlation of m6A RNA methylation with MTDH might contribute to the mechanism of MTDH in immunotherapy resistance. Furthermore, our analysis illustrated a positive mutual correlation between MTDH expression, EMT score, and m6A RNA methylation in pan-cancer levels, showing a molecular axis from MTDH to m6A RNA methylation and EMT existed in cancer samples.

Taking the above knowledge together with the protein-protein interaction network between MTDH, m6A RNA methylation, and EMT established in this study, the comprehensive signaling from MTDH to m6A RNA methylation and EMT in order could be a potential mechanism underlying immunotherapy resistance. Moreover, we explored the MTDH-m6A-RNA-methylation-EMT-associated gene signature in cancers. M2 macrophages, Th2 cells, and fibroblasts were confirmed to be positively correlated with the MTDH-m6A-RNA-methylation-EMT-associated gene signature, while CD4^+^ Tcm, NK cells, and Th1 cells negatively correlated with this comprehensive signature. Therefore, it would be expected that the MTDH-m6A-RNA-methylation-EMT-associated gene signature would influence immunotherapy response. The higher estimated score of MTDH-m6A-RNA-methylation-EMT-associated gene signature was founded in non-responders or worse overall survival patients treated by immunotherapy, which confirmed the clinical importance of checking this comprehensive signature in the tumor before given immunotherapy.

In conclusion, we systematically investigated the expression of MTDH and its associations with cancer prognosis, immunotherapy response, tumor-infiltrating cells, and immune checkpoints in multiple independent cohorts. m6A RNA methylation and EMT may involve the associations of MTDH with immunotherapy resistance and cancer prognosis. Although more designed experiments and trials need to be performed, the development of blockades to MTDH and immune checkpoints on tumors may help cancer cells overcome the primary or adaptive resistance to immunotherapy and repress its metastasis, resulting in the long-lasting treatment effect of immunotherapy on a broader range of patients with cancer.

### Limitation of the study

Since this is a retrospective pan-cancer study, the potential bias from different cohort patients or clinical trials is inevitable, which might confound our results to some extent. As far as we know, there is no standardized definition of immunotherapy responders on the stable disease. In other words, there is no consensus that whether stable disease with six months duration or with 12 months should be considered as a responder. Here, the definition of responders in our included immunotherapy clinical trials also shows a bit of variance when including the patients with stable disease, which might bring an unknown bias to our analysis. Furthermore, the findings in this study were confirmed in multiple cancer RNA/protein profiles and clinical trials datasets by in silico analysis. More clinical trials or large population cohorts are needed to validate the possible translational role of MTDH in cancer immunotherapy.

## STAR★Methods

### Key resources table

**Table:** 

REAGENT or RESOURCE	SOURCE	IDENTIFIER
**Deposited data**		

Pan-Cancer RNA expression Data	TCGA	https://portal.gdc.cancer.gov; https://xenabrowser.net/
Clinical Data for TCGA Cohorts	Thorsson et al., 2018	Table S1
Independent Cancer Cohort 1	Wichmann et al., 2015	GEO: GSE65858
Independent Cancer Cohort 2	Yamauchi et al., 2012	GEO: GSE31210
Independent Cancer Cohort 3	Kao et al., 2011	GEO: GSE20685
Cancer Immunotherapy Cohort 1	Auslander et al., 2018	GEO: GSE115821
Cancer Immunotherapy Cohort 2	Cho et al., 2020	GEO: GSE126044
Cancer Immunotherapy Cohort 3	Riaz et al., 2017	https://github.com/riazn/bms038_analysis
Cancer Immunotherapy Cohort 4	Van Allen et al., 2015	dbGap: phs000452.v2.p1

**Software and algorithms**		

clusterProfiler	Yu et al., 2012	https://guangchuangyu.github.io/software/clusterProfiler
xCell	Aran et al., 2017	https://xcell.ucsf.edu
MCP-Counter	Becht et al., 2016	https://github.com/ebecht/MCPcounter
TIMER	Li et al., 2017b	http://cistrome.org/TIMER
CIBERSORT	Newman et al., 2015	https://cibersort.stanford.edu
String	Szklarczyk et al., 2019	https://string-db.org
Cytoscape (v3.8.2)	Shannon et al., 2003	https://cytoscape.org
R -project	R Foundation for Statistical Computing	R version 4.0.1
R codes in this study	This paper	https://github.com/entcai/MTDH_immunology

### Resource availability

#### Lead contact

Further information and requests for resources and reagents should be directed to the lead contact, GC Zhu (qianhudoctor@csu.edu.cn).

#### Materials availability

This study did not generate new unique reagents.

### Methods details

#### Datasets and cohorts

The pan-Cancer normalized gene-level RNA-Seq data for 33 TCGA cohorts were downloaded from UCSC Xena (https://xenabrowser.net/). Only Primary Tumor and matched normal tissues were saved for further analysis. Clinical data for TCGA were download from a TCGA-Clinical Data Resource (CDR) (Thorsson et al., 2018). The tumor mutation burden (TMB) and neoantigen number in TCGA were acquired from Thorsson et al.(Thorsson et al., 2018). The available cohorts (Independent Cancer Cohort 1-3) for validations were downloaded from the GEO database (head and neck cancer: GSE65858 (Wichmann et al., 2015), lung adenocarcinoma: GSE31210 (Yamauchi et al., 2012); invasive breast cancer: GSE20685 (Kao et al., 2011)) and an online platform (Chen et al., 2019) based on the NCI Clinical Proteomic Tumor Analysis Consortium (CPTAC) database including breast cancer, ovarian cancer, colon cancer, clear cell renal cell carcinoma, Uterine corpus endometrial carcinoma, lung adenocarcinoma, and pediatric brain cancer.

The pooled objective response rate for immune checkpoint blockade in different types of cancer in the TCGA dataset has been described previously (Yarchoan et al., 2017) and updated in a recent study (Wang et al., 2019). The validations of cancer immunotherapy cohorts (Cancer Immunotherapy Cohort 1-4) were retrieved from four independent datasets with RNA-seq data (Auslander et al., 2018; Cho et al., 2020; Riaz et al., 2017; Van Allen et al., 2015). The Van Allen et al. (Science, 2015) dataset (Van Allen et al., 2015) related to CTLA-4 blockade in metastatic melanoma and defined ‘clinical benefit’ using a composite endpoint of complete response or partial response to CTLA-4 blockade as assessed by RECIST criteria or stable disease by RECIST criteria with overall survival greater than one year, ‘no clinical benefit was defined as a progressive disease by RECIST criteria or stable disease with overall survival less than one year. The Noam Auslander et al. (Nat Med, 2018) dataset (Auslander et al., 2018) related to anti-PD1 or anti-CTLA4 therapy in metastatic melanoma: responding tumors were derived from patients who have complete or partial responses; non-responding tumors were derived from patients who had progressive disease. The Nadeem Riaz et al. (Cell, 2017) dataset (Riaz et al., 2017) related to PD-1 blockade in melanoma: responding tumors were derived from patients who have complete or partial responses; non-responding tumors were derived from patients who had progressive disease or stable disease. The Jae−Won Cho et al. (Exp Mol Med, 2020) dataset (Cho et al., 2020) related to PD-1 blockade in non-small cell lung cancer: responding tumors were derived from patients who have complete or partial responses or stable disease for >6 months; otherwise, the patient would be defined as non-responders. RNA-Seq data in these cohorts were used to calculate the MTDH expression and MTDH-m6A-RNA-methylation-EMT signature for each patient. The median value is used as the threshold to separate the high and low groups.

#### Immune cell deconvolution and signature estimation on gene set

The proportions of tumor-infiltrating immune and stromal cell populations in TCGA cancer were deconvoluted from corresponding bulk RNA sequencing data by xCell (Aran et al., 2017), MCP-Counter (Becht et al., 2016), TIMER (Li et al., 2017b), and CIBERSORT (Newman et al., 2015) algorithms. The estimated scores of EMT, m6A RNA methylation, and the comprehensive pathway of MTDH-m6A-RNA-methylation-EMT were evaluated by principal component analysis and Z-score by IOBR package (Lee et al., 2008; Zeng et al., 2020). EMT associated gene set includes SOX9, TWIST1, FOXF1, ZEB1, ZEB2, and GATA6 (Mariathasan et al., 2018). METTL3, METTL14, RBM15, RBM15B, WTAP, KIAA1429, CBLL1, ZC3H13, ALKBH5, FTO, YTHDC1, YTHDC2, YTHDF1, YTHDF2, YTHDF3, IGF2BP1, HNRNPA2B1, HNRNPC, FMR1, LRPPRC, and ELAVL1 were calculated for the m6A RNA methylation signature (Zhang et al., 2020). The comprehensive pathway of MTDH-m6A-RNA-methylation-EMT consists of genes in the EMT-associated gene set, m6A RNA methylation gene set, and MTDH.

#### GO, KEGG enrichment assay and protein-protein interaction network

The differential gene expression (DGE) analysis was applied to compare the variable genes between MTDH-high and MTDH-low groups by the limma package. Genes with p-value < 0.01, FDR < 0.05 and absolute value of log2FC >1 would be considered as DGE genes and inputted in GO and KEGG pathway enrichment analysis by using the clusterProfiler package (Yu et al., 2012). GO terms and KEGG pathways with an adjusted p-value less than 0.05 were considered to be statistically significant. For enrichment analysis, the DGE genes were inputted into enricher function in the clusterProfiler package (Yu et al., 2012).

The protein-protein interaction (PPI) network between MTDH, EMT, and m6A RNA methylation was constructed by String online web (Szklarczyk et al., 2019) and visualized by Cytoscape (v3.8.2) (Shannon et al., 2003) after inputting the DGE genes and the gene set in EMT and m6A RNA methylation.

### Quantification and statistical analysis

A pairwise student t-test was used to compare the MTDH expression in tumors and matched adjacent normal tissues. Kaplan-Meier analysis or multivariate cox analysis was performed for survival analysis. The Pearson coefficient was applied to analyze correlations. Wilcox test was used to compare the difference between the two variables. Meta-analysis with the random effect model was applied to test the pooled effect of MTDH expression on immunotherapy response in four cohorts with immune checkpoint blockade treatment. All reported p-values are two-tailed, and for all analyses, p < 0.05 is considered statistically significant unless otherwise specified. Statistical analyses were performed using R (version 4.0.1).

### Additional resources

No additional resource was used in this study.

## Data Availability

• This paper analyzes existing, publicly available data. These accession numbers for the datasets are listed in the key resources table. • R codes used in this study have been deposited and publicly available at GitHub. DOIs are listed in the key resources table. • Any additional information required to reanalyze the data reported in this paper is available from the lead contact upon request.
