# Supplementary material for: MTDH associates with m6A RNA methylation and predicts cancer response for immune checkpoint treatment
Source: iScience. 2021 Sep 9;24(10):103102. doi: 10.1016/j.isci.2021.103102 (PMC8479698; doi:10.1016/j.isci.2021.103102)
Supplement: Document S1. Figures S1–S4 [file mmc1.pdf]

iScience, Volume *24*

Supplemental information

MTDH associates with m6A RNA methylation and predicts cancer response for immune checkpoint treatment

Fen Zhang, Huimei Huang, Yuexiang Qin, Changhan Chen, Li She, Juncheng Wang, Donghai Huang, Qinglai Tang, Yong Liu, Gangcai Zhu, and Xin Zhang

Figure S1

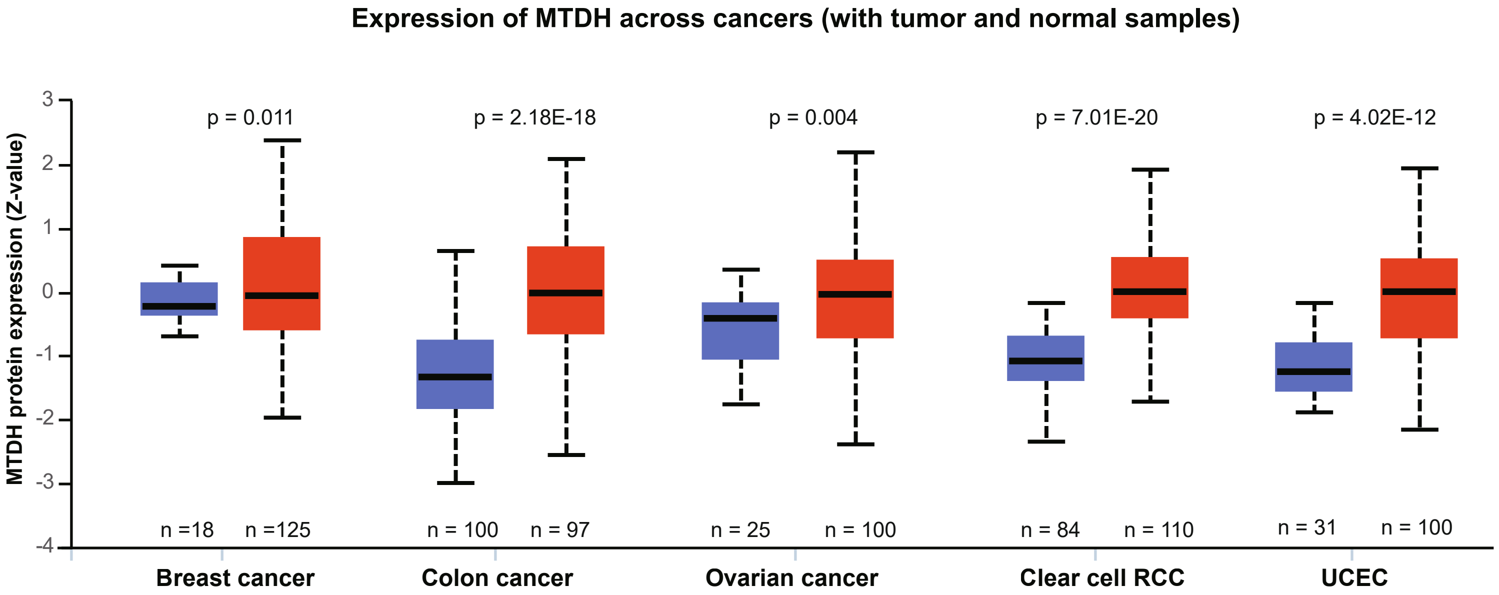

Figure S1. MTDH protein expression is significantly higher in tumors than adjacent normal tissues across different types of cancer, Related to Figure 1.

Figure S2

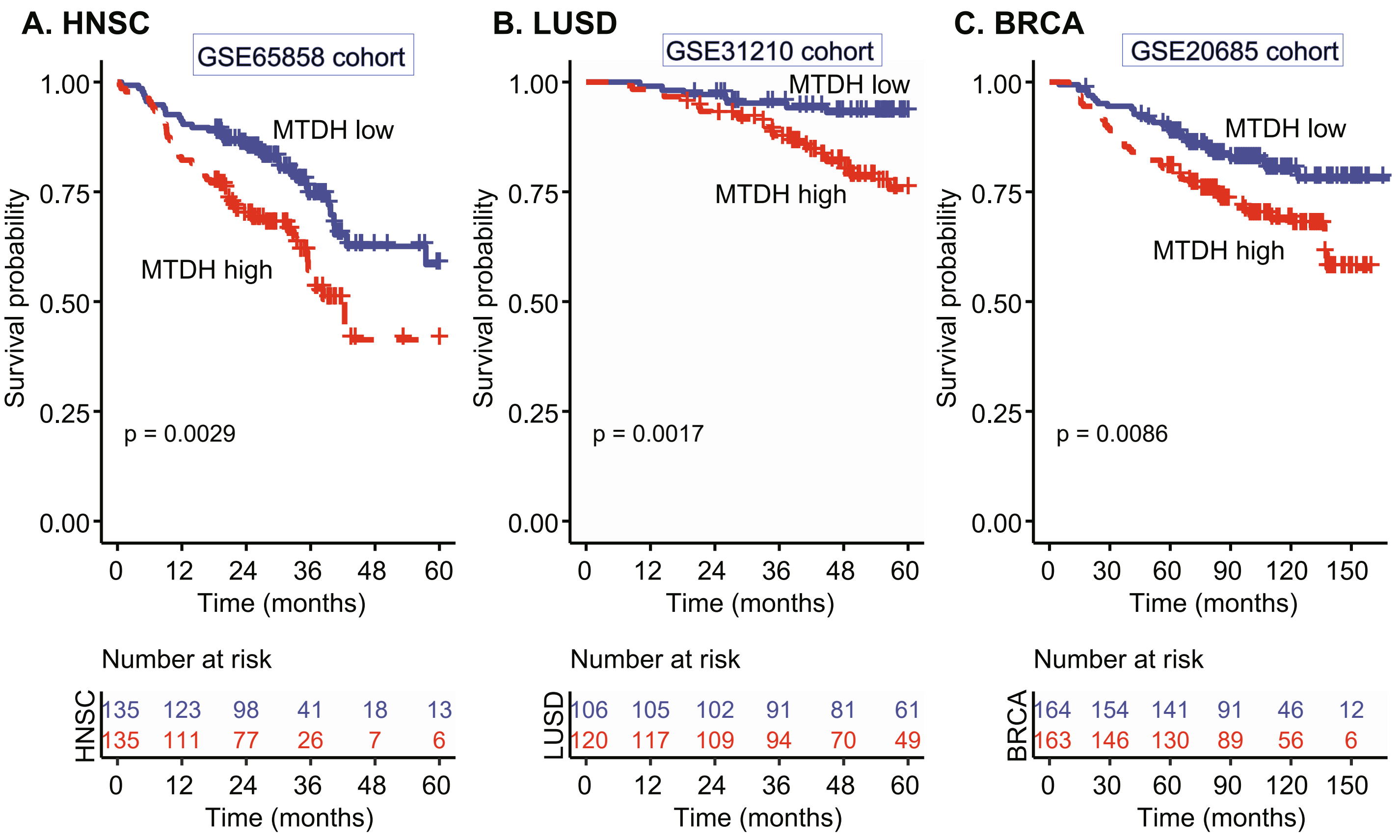

Figure S2. High expression of MTDH associates with a poor overall survival in across cancers, Related to Figure 2.
(**A-C**): Kaplan-Meier survival analysis displaying in overall survival between cancer patients with high and low MTDH expression.
LUAD: Lung adenocarcinoma, **BRCA:** Breast invasive carcinoma, **HNSC:** Head and Neck squamous cell carcinoma.

Figure S3

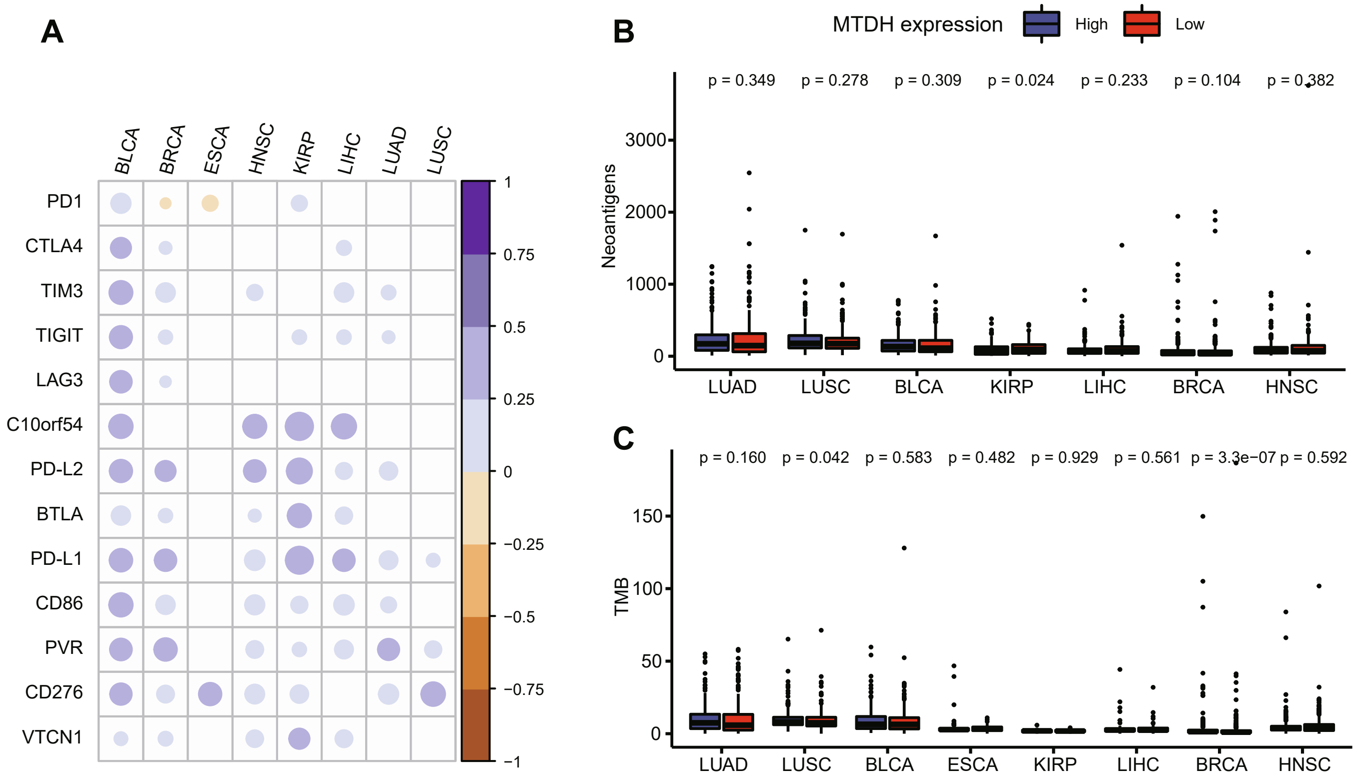

Figure S3. The correlation of MTDH expression with immune chekpoints, TMB, and neoantigens, Related to Figure 4.
(A): the colored cycles shows the pearson correlations of MTDH expression with immune checkpoints in BLCA, BRCA, ESCA, HNSC, KIRP, LIHC, LUAD, and LUSC; the cycled block presents into white if p value less than 0.05. the depth of color represents the extent of pearson correlation;
(B): There is no significant difference of neoantigen number between tumors with MTDH high-expression and MTDH low-expression cancer.
(C): There is no significant difference of tumor mutation burden (TMB) between MTDH high-expression and MTDH low-expression groups in most of cancers.
BLCA: Bladder Urothelial Carcinoma, **ESCA:** Esophageal carcinoma, **KIRP:** Kidney renal papillary cell carcinoma, **LUAD:** Lung adenocarcinoma, **BRCA:** Breast invasive carcinoma, **HNSC:** Head and Neck squamous cell carcinoma, **LIHC:** Liver hepatocellular carcinoma, **LUSC:** Lung squamous cell carcinoma.

Figure S4

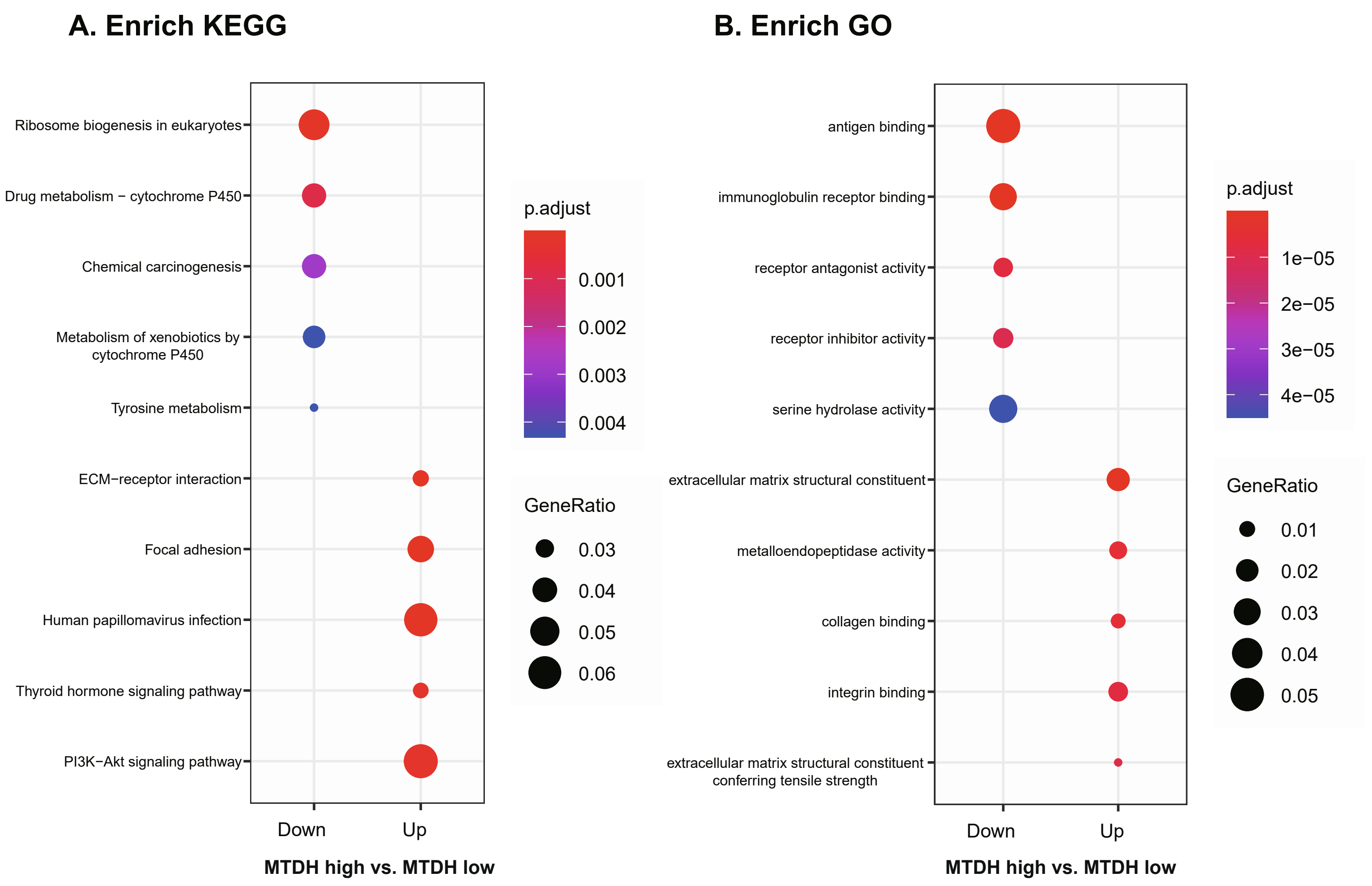

Figure S4. GO function and KEGG pathway enrichment assay for the differential expressed genes comparing tumors with high expression of MTDH to tumors with low expressed MTDH, Related to Figure 5.
